# Fast-growing Arctic Fe–Mn deposits from the Kara Sea as the refuges for cosmopolitan marine microorganisms

**DOI:** 10.1038/s41598-022-23449-6

**Published:** 2022-12-20

**Authors:** Natalia Shulga, Sergey Abramov, Alexandra Klyukina, Konstantin Ryazantsev, Sergey Gavrilov

**Affiliations:** 1 Shirshov Institute of Oceanology, Russian Academy of Sciences, Moscow, Russia; 2 Department of Environmental Microbiology, Institute of Sanitary Engineering, Water Quality and Solid Waste Management, University of Stuttgart, Stuttgart, Germany; 3 Winogradsky Institute of Microbiology, Research Center of Biotechnology, Russian Academy of Sciences, Moscow, Russia; 4 Vernadsky Institute of Geochemistry and Analytical Chemistry, Russian Academy of Sciences, Moscow, Russia

**Keywords:** Marine microbiology, Geochemistry

## Abstract

The impact of biomineralization and redox processes on the formation and growth of ferromanganese deposits in the World Ocean remains understudied. This problem is particularly relevant for the Arctic marine environment where sharp seasonal variations of temperature, redox conditions, and organic matter inflow significantly impact the biogenic and abiotic pathways of ferromanganese deposits formation. The microbial communities of the fast-growing Arctic Fe–Mn deposits have not been reported so far. Here, we describe the microbial diversity, structure and chemical composition of nodules, crust and their underlying sediments collected from three different sites of the Kara Sea. Scanning electron microscopy revealed a high abundance of microfossils and biofilm-like structures within the nodules. Phylogenetic profiling together with redundancy and correlation analyses revealed a positive selection for putative metal-reducers (*Thermodesulfobacteriota*), iron oxidizers (*Hyphomicrobiaceae* and *Scalinduaceae*), and Fe-scavenging *Nitrosopumilaceae* or *Magnetospiraceae* in the microenvironments of the Fe–Mn deposits from their surrounding benthic microbial populations. We hypothesize that in the Kara Sea, the nodules provide unique redox-stable microniches for cosmopolitan benthic marine metal-cycling microorganisms in an unsteady environment, thus focusing the overall geochemical activity of nodule-associated microbial communities and accelerating processes of ferromanganese deposits formation to uniquely high rates.

## Introduction

In recent years, deep-sea ferromanganese (Fe–Mn) nodules and crusts have attracted considerable attention as a potential source of iron and manganese, as well as other technology metals and rare earth elements (REE)^1–3^. Wide and dense ore fields occur mainly in the regions with very low sedimentation rates (pelagic areas and submarine outcrops of the Pacific and Eastern Indian Oceans). Polymetallic deposits slowly grow there at a rate of several mm to several cm per million years, depending on their origin^3^. Several suggestions have been made on the mode of origin of ferromanganese nodules, depending on their metal sources. Those included hydrogenetic precipitation of metals from cold ambient water, oxic/suboxic diagenesis from sediment-pore fluids, or precipitation of Fe–Mn oxyhydroxides directly from hydrothermal solutions^4–8^. However, the current view on the genesis of Fe–Mn deposits leaves out the processes leading to rapid formation of the Fe–Mn nodules and crusts in shallow-water regions. These ‘shallow-water’ deposits are mainly located at depths of down to 300 m and grow about 10^3^ times faster than their deep-sea counterparts^9^.

**Figure 1:**
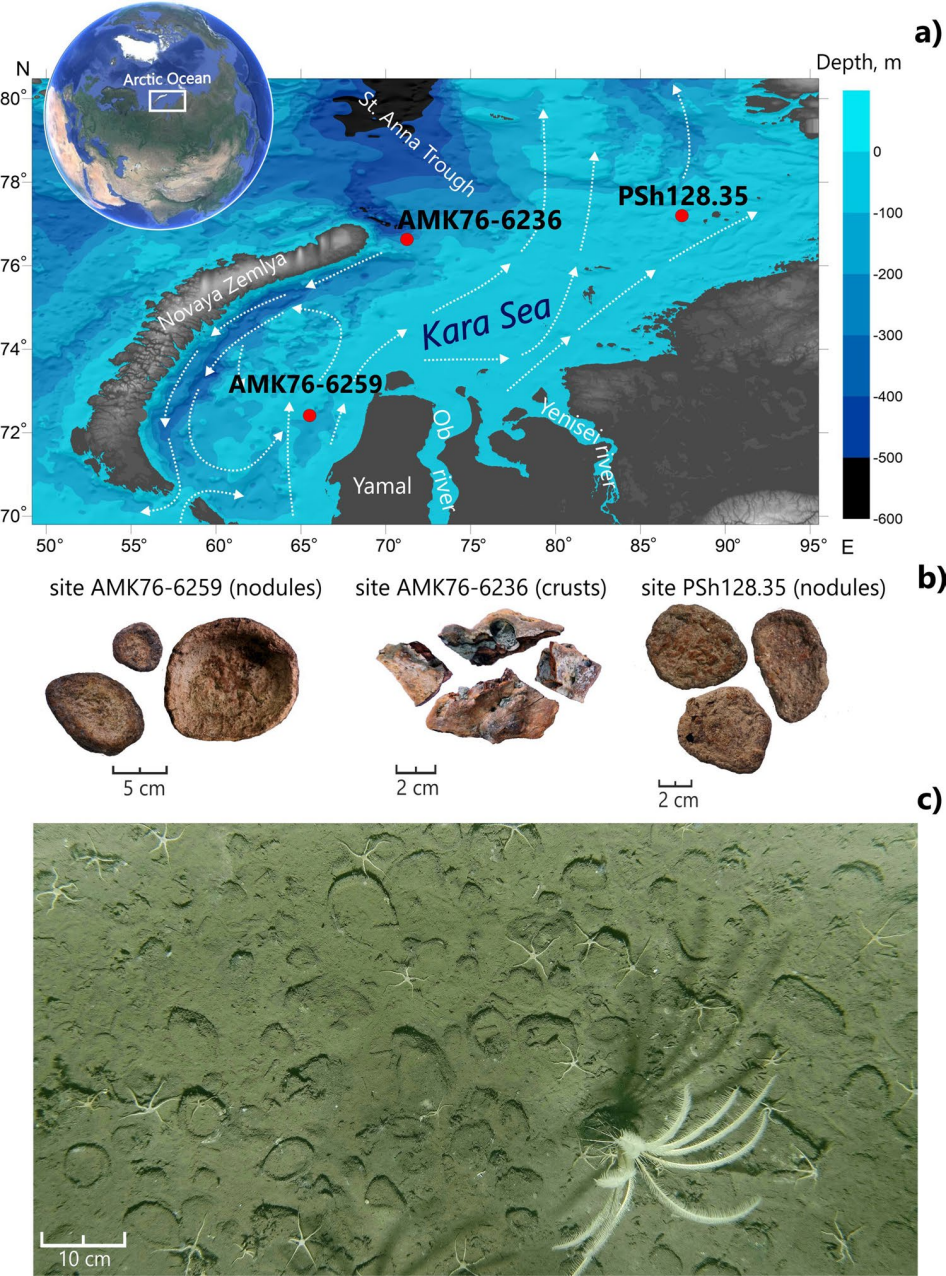
(**a**) The map of sampling sites location and circulation of surface currents (*from Stein*^29^). (**b**) General view of the studied Fe–Mn deposits. (**c**) Fe–Mn nodules on site AMK76-6259 covered by a fluffy layer (sea-floor photo by Sonar ocean bottom surveying Lab, IO RAS).

Along with the generally accepted view on the genesis of the Fe–Mn nodules and crusts as a combination of different geochemical processes, an increasing amount of evidence is now accumulating in favor of microbial activity involvement in ferromanganese deposits formation^10–20^. As the major players of diagenesis, microorganisms can influence iron and manganese mineralization. There are prokaryotes able to oxidize Fe^2+^ and Mn^2+^ for using it as a source of energy, and microorganisms capable of reducing Fe^3+^ and Mn^4+^ as terminal electron acceptors in electron transfer chains^21,22^. The three basic processes of biological control (structural, spatial and chemical) over mineralization^23^ were thoroughly described back in the 1980s. However, the information about the exact microbial groups that drive biomineralization has only become available with the development of molecular phylogenetics and ecology. Recent studies based on 16S rRNA gene profiling of microbial communities from the deep-sea (various sites of Pacific Ocean) and shallow-water (Baltic Sea) Fe–Mn nodules, as well as from the Atlantic deep-sea Fe–Mn crusts, provided the first insights into the structure and possible functions of microbial populations inhabiting the ferromanganese deposits of the World Ocean. The communities of the nodules revealed a lower phylogenetic diversity compared to that reported for underlying sediments. At the same time, the reported nodules communities were enriched with one or several taxa represented in both microenvironments^11,13,14,16,24–28^. Notably, the microbial communities of any Fe–Mn deposits from the Arctic Ocean have not been characterized so far. As a shallow marginal shelf-sea of the Arctic Ocean, Kara Sea is considered an important reference point for the understanding of global biogeochemical cycles. In this work, we describe the phylogenetic composition of microbial communities associated with the Arctic ferromanganese nodules and crusts collected from geographically distant sites of the Kara Sea (Fig. 1), characterize their chemical parameters, and discuss the geochemical and ecological aspects of presumed microbe-to-mineral interactions in the view of their involvement in iron and manganese mineralization within the contrasting polar environment.

## Results

### Bulk geochemistry of nodules, crust and sediments

Major and trace element abundances and their comparative ratios in three bulk samples of Fe-Mn deposits and underlying sediments are presented in Table 1. The concentration of Mn, Fe, Co, Ni, Mo, and P is 4- to 57-fold higher in nodules than in the underlying sediments. The nodules are enriched in Mn and Mo. In general, the analyzed mineral deposit samples could be divided into two groups (Fe-rich and Mn-rich) according to their geochemical characteristics. The Mn/Fe ratio in the Fe-rich crust and nodule (from sites AMK76-6236 and PSh128.35, respectively) reaches 0.01 and 0.32, respectively, while in the Mn-rich nodule (site AMK76-6259) this ratio value reaches 1.65. The abundance of major and trace elements in underlying sediments was generally similar at all the sampling sites. The exception is the Mn content which is  4-8 times lower in sediments from the Saint Anna Trough (site AMK76-6236) compared to the other sites. The concentration of Al is higher in the sediments than in the nodules and crusts. It is generally caused by a higher content of clay minerals and detritus in underlying sediments in comparison to nodules. The total REE and yttrium (REY) concentrations are low (139–181 ppm) and almost similar in all the studied samples of sediments and mineral deposits. The nodules are depleted of light REE and enriched in heavy REE (LREE_NASC_/HREE_NASC_ 0.86–0.87) and vice versa for the crust. A negative Ce anomaly and a positive Y anomaly were detected in all samples.

**Table 1:** Element composition of nodules, crust and underlying sediments from the Kara Sea.

Elements	Sampling site					
	AMK76-6259		PSh128.35		AMK76-6236	
	Nodule	Sediments	Nodule	Sediments	Crust	Sediments
Fe (wt%)	11.5	2.79	24.4	3.78	17.6	4.27
Mn	18.9	0.33	7.80	0.18	0.21	0.04
Al	2.77	4.94	2.53	5.09	3.87	7.29
Ca	1.16	0.72	1.50	0.68	0.95	0.69
K	1.22	2.13	1.06	1.72	1.63	2.55
Mg	1.07	0.71	0.96	0.86	0.81	1.43
Na	1.96	2.13	1.68	1.82	1.18	2.00
P	0.65	0.07	1.58	0.09	0.83	0.10
S	0.11	0.10	0.13	0.11	0.05	0.16
Ti	0.19	0.38	0.17	0.33	0.25	0.46
As (ppm)	396	33.1	952	45.3	523	18.9
Ba	974	542	487	577	392	811
Be	0.66	1.20	0.94	1.40	1.07	2.04
Bi	0.10	0.12	0.10	0.13	0.12	0.18
Cd	0.81	< 0.07	1.21	0.04	0.17	< 0.07
Co	181	13.5	164	16.3	17.7	12.2
Cr	40.3	46.8	37.7	59.7	37.6	80.7
Cs	2.01	2.67	1.57	3.10	2.23	5.95
Cu	42.2	12.3	23.0	15.8	9.68	20.3
Ga	27.3	9.52	13.7	12.2	7.75	16.6
Hf	1.11	1.96	1.03	1.90	1.36	3.23
Li	205	18.0	57.6	24.6	18.5	47.3
Mo	250	5.66	172	3.80	7.27	1.40
Nb	4.10	7.75	3.76	7.60	5.10	9.09
Ni	163	24.3	167	29.1	16.8	40.1
Pb	12.0	16.0	17.0	14.3	26.6	19.5
Rb	39.1	70.1	29.8	77.2	53.6	108
Sb	30.8	1.13	10.9	1.20	2.17	0.70
Sc	5.04	8.98	5.02	10.4	6.40	16.79
Sn	0.40	0.84	0.39	1.00	0.51	1.59
Sr	562	178	734	194	369	126
Ta	0.24	0.50	0.24	0.55	0.29	0.61
Th	3.27	11.57	3.00	5.40	5.12	8.87
Tl	4.95	0.41	2.59	0.38	0.55	0.66
U	8.27	1.40	24.7	1.40	8.75	2.68
V	267	124	577	142	251	175
W	8.64	1.14	5.16	1.10	1.18	1.14
Y	26.5	14.7	35.1	14.1	18.0	19.4
Zn	95.1	47.2	100	60.7	69.9	87.8
Zr	53.5	70.2	53.4	73.4	58.4	116
La	22.6	25.3	31.8	24.1	25.6	27.8
Ce	40.2	55.5	47.0	51.5	50.5	60.2
Pr	5.13	6.20	6.79	5.50	5.89	6.80
Nd	21.7	24.3	29.4	21.9	24.1	27.7
Sm	4.81	4.70	6.42	4.20	4.75	5.60
Eu	1.16	1.05	1.48	1.00	1.05	1.16
Gd	5.16	3.81	6.83	3.90	4.12	4.51
Tb	0.72	0.53	0.99	0.57	0.56	0.64
Dy	4.38	3.21	6.13	3.00	3.42	3.70
Ho	0.98	0.60	1.28	0.59	0.65	0.76
Er	2.73	1.74	3.69	1.70	1.91	2.29
Tm	0.37	0.24	0.48	0.24	0.26	0.33
Yb	2.48	1.59	3.29	1.70	1.76	2.32
Lu	0.36	0.23	0.50	0.24	0.26	0.35
∑ REY	139	144	181	134	143	163
Co + Ni + Cu (ppm)	386	50.1	354	61.2	44.2	72.6
Mn/Fe	1.65	0.12	0.32	0.05	0.012	0.01
Ce_anomaly_	0.81	0.96	0.70	0.97	0.90	0.95
LREE/HREE	0.86	1.58	0.87	1.38	1.38	1.22

Ce_anomaly_ = (2 × CeSN)/(LaSN + NdSN).

LREE/HREE = (LaSN + 2 × PrSN + NdSN)/(ErSN + TmSN + YbSN + LuSN).

*SN* shale normalised.

### Internal structure of nodules

**Figure 2:**
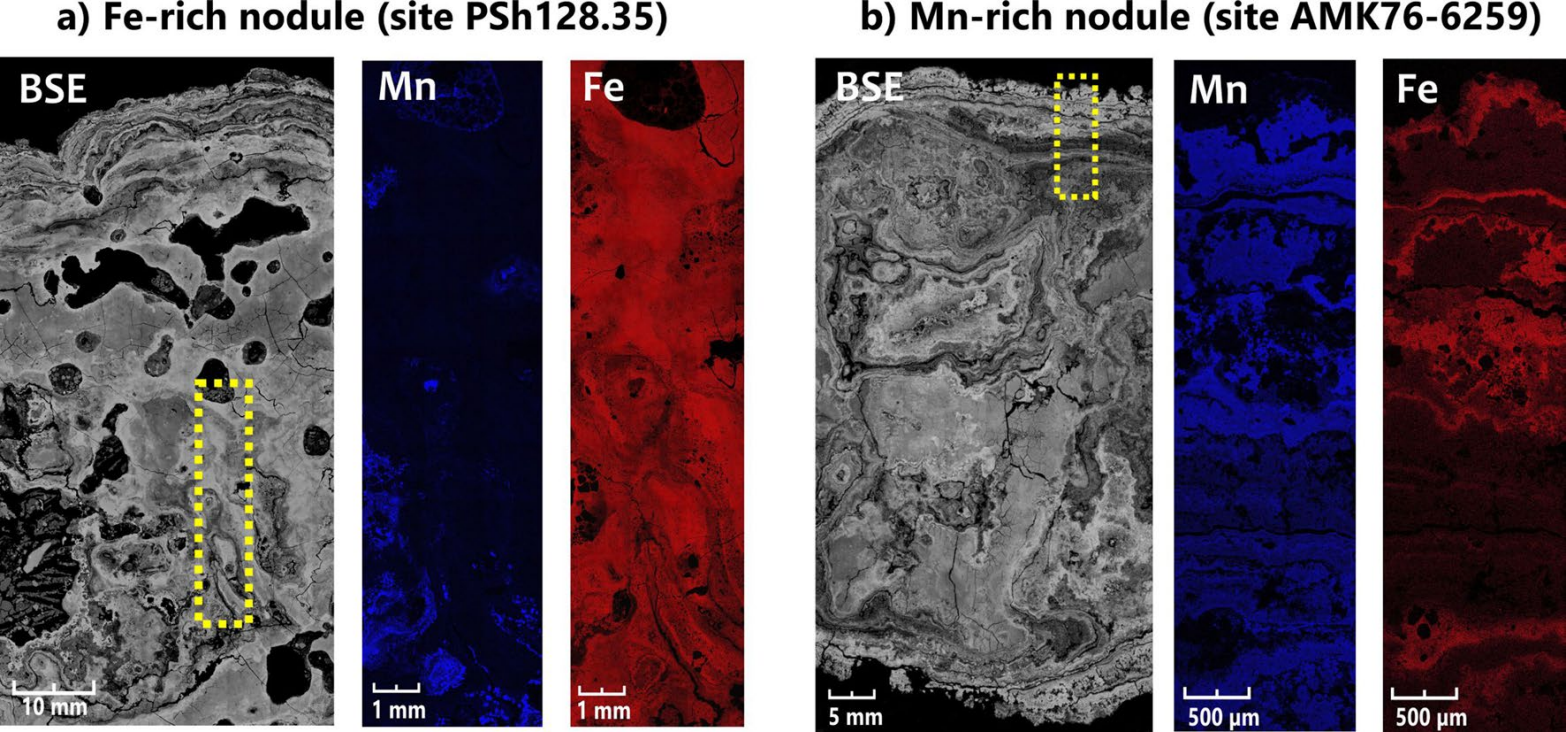
Growth structures and elemental map in nodule samples from sites PSh128.35 and AMK76-6259. Yellow dotted rectangles show the areas of elemental mapping (Mn and Fe).

Scanning electron microscopy (SEM) and backscattered electron imaging (BSE) of polished thin-sections demonstrate that nodules have different growth patterns with well-distinguished concentric outermost layers and more complex and irregular layers of the interior. Furthermore, clear differences between the Fe-rich and Mn-rich nodules were revealed using elemental mapping (Fig. 2). The distribution of Fe and Mn is characterized by antiphase, particularly in microbotryoids. The nodule and crust samples also contain a substantial amount of angular aluminosilicate detrital material.

**Figure 3:**
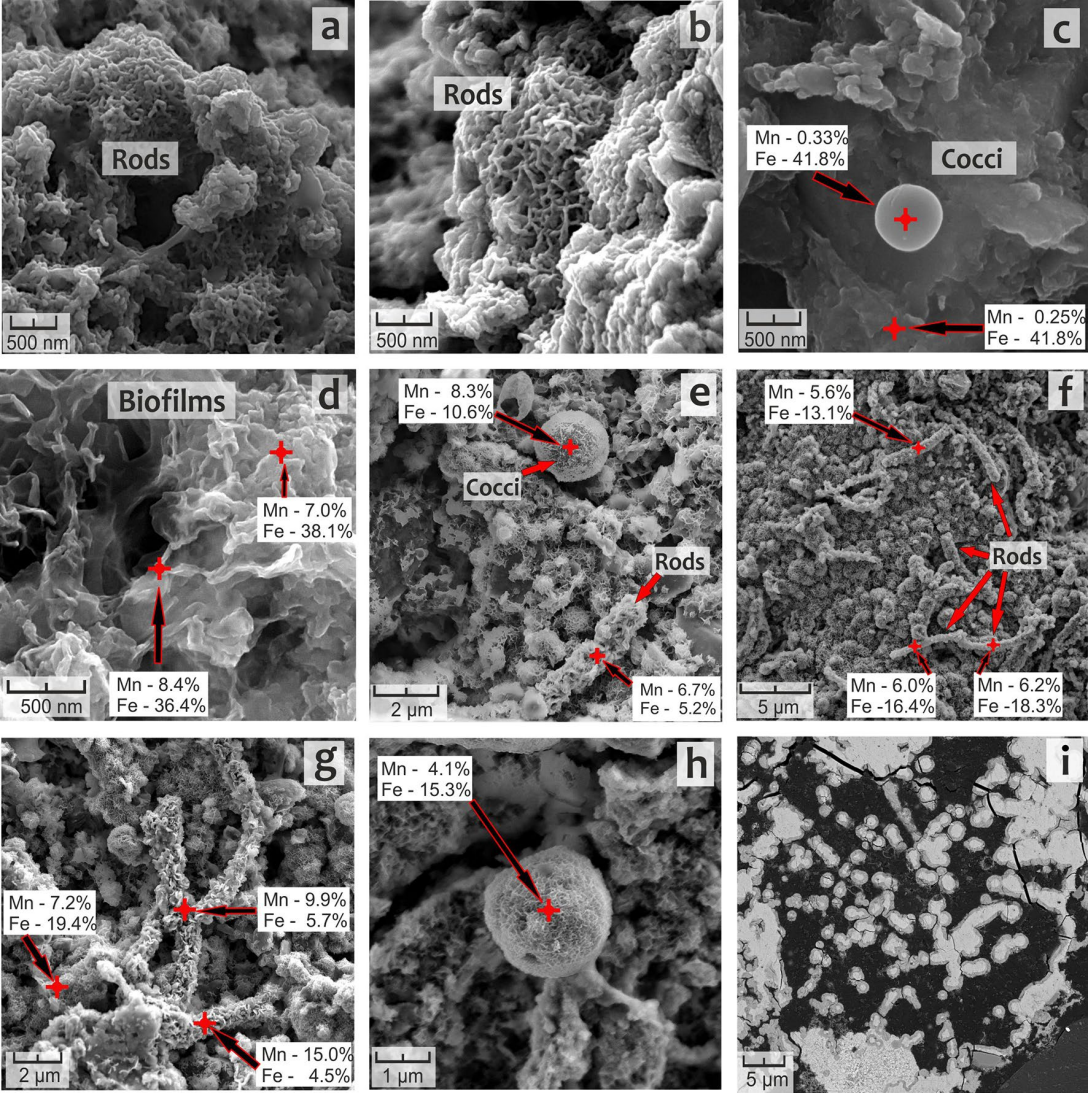
SEM images of (**a–d**) Fe-rich nodule (site PSh128.35) (central part of the nodule); (**e–h**) Mn-rich nodule (site AMK76-6259) (central part of the nodule); (**i**) laminated microtexture surrounding the globular structures which have the dimensions of coccoid microfossils.

SEM revealed structures similar to microbial cells and biofilms (Fig. 3) in the nodule’s interior. Within the inner part of the Fe-rich nodule from site PSh128.35, these cell-like microfossils (rods- and cocci-shaped) and biofilm-like fossils have smooth surfaces lacking visual signs of mineralization (Fig. 3a–d). Coccoid particles have a diameter of 300 to 500 nm and are located separately from the rod-shaped ones. Rods are more abundant and have a length of 200–400 nm, and a width of 50–70 nm. Individual rods are distributed chaotically or combined in ‘colonies’ (Fig. 3a,b). The material surrounding the cell-like fossils is of homogeneous solid matter (Fig. 3c). Crystalline texture of the surface of the fossils and the underlying matter between them was observed in the Mn-rich nodule from site AMK76-6259 (Fig. 3e–h). The mineralized cell-like microfossils from this site are larger than the others. The diameter of coccoid particles reaches 2 µm, while the length of rod-shaped particles varies from 2.5 to 5 µm. Energy-dispersive X-ray spectroscopy (EDS) of central zones of the nodules has revealed that cell-like fossils and biofilms in the sample from site PSh128.35 are predominantly encrusted with Fe-containing minerals (Fig. 3c,d). In contrast, the fossils from the Mn-rich sample (site AMK76-6259) are encrusted with both Fe- and Mn-containing minerals (Fig. 3e–h).

### Content of n-alkanes in nodules and crust

The total organic carbon (TOC) content in nodules and crust reaches 0.41%, 0.99% and 0.57%, respectively, and is slightly lower than in the underlying sediments (1.2–1.4%). N-alkanes concentration (n-C_13_-C_33_) in nodules and crust varies from 0.96 to 1.24 µg/g of dry weight, which is  comparable to the n-alkanes content in the sediments (Table S1). High molecular weight n-alkanes (HMW, > C_23_) with a C_max_ at C_27_ have the highest proportions in the Mn-rich nodule (site AMK76-6259) and the Fe-rich crust (site AMK76-6236). In contrast, n-alkanes fraction of the Fe-rich nodule (site PSh128.35) shows bimodal distribution of homologs with an increased proportion of low molecular weight (LMW) ones having a C_max_ of C_16_ and C_18_. Carbon preference indices of n-alkanes from both nodules and from the crust are high for HMW fractions (CPI > 5) and low for LMW fractions (OEP_17-19_ < 0.5). The underlying sediments are characterized by a unimodal distribution of n-alkanes on site AMK76-6259 and bimodal distribution on site AMK76-6236. The values of CPI and OEP_17-19_ indexes similar to those determined for the Fe–Mn deposits.

### Microbial communities colonizing Fe–Mn deposits and underlying sediments

**Figure 4:**
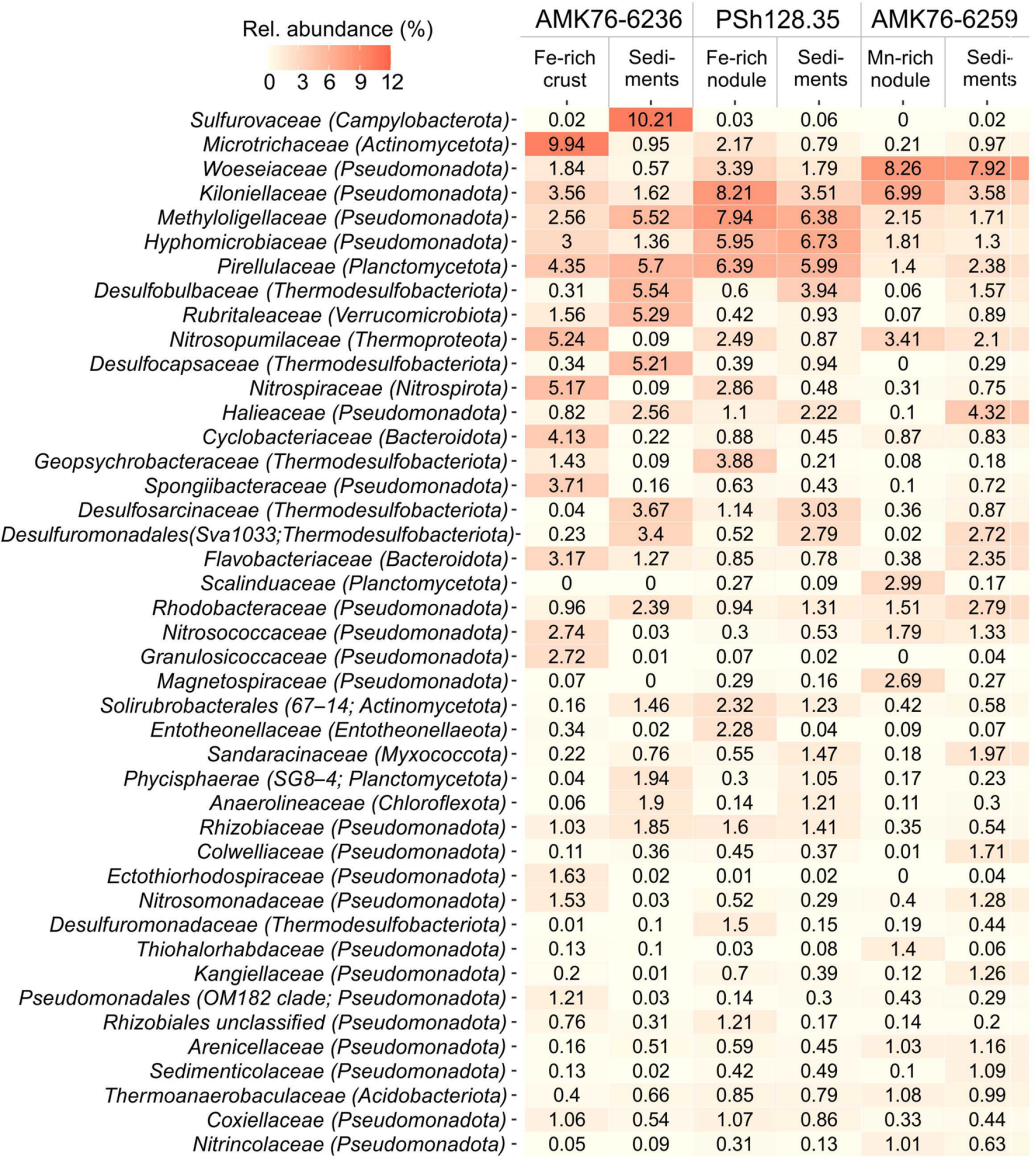
Heatmap illustrating relative 16S rRNA gene sequence abundance (at family level; > 1%) of nodule-, crust-, and sediment-associated microbial communities sampled at sites AMK76-6236, PSh128.35 and AMK76-6259. Relative abundances of specific taxa represent average value of duplicate samples (Tables S3).

**Figure 5:**
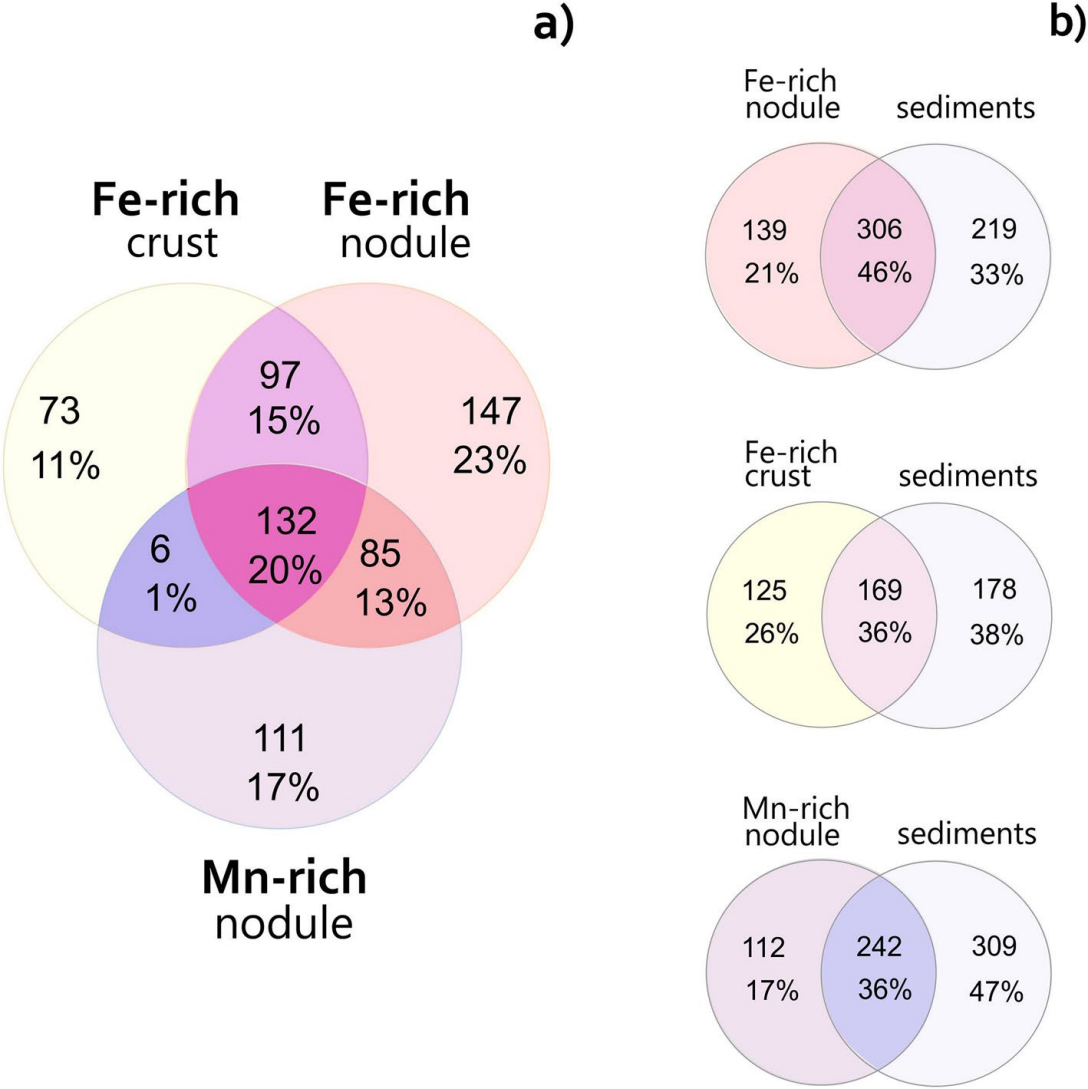
Venn diagram showing the distribution of unique OTUs and the number of shared OTUs (overlapped regions) between Fe-rich crust (site AMK79-6236), Fe-rich nodule (site PSh128.35) and Mn-rich nodule (site AMK79-6259); **(a)** comparison of three different Fe-Mn deposits; **(b)** pairwise comparison of the Fe-Mn deposits and their underlying sediments.

We identified 16S rRNA gene relative abundances of prokaryotic taxa in microbial communities of the Fe–Mn nodules, crust and their underlying sediments at all the sampling sites (Fig. 4). Each of the identified microbial communities contains unique and shared operational taxonomic units (OTUs) (Fig. 5), representing bacterial and archaeal families unevenly distributed in the Fe–Mn deposits and the sediments.

The microbial communities of the Fe–Mn deposits from all three sites share a rather high proportion of their diversity (ca. 20% OTUs). The taxa shared between the Fe-rich and Mn-rich nodules, or between the Fe-rich nodule and crust comprise smaller proportions (Fig. 5a). Pairwise comparison of community compositions of the Fe–Mn deposits and their underlying sediments revealed substantial proportions of phylotypes inhabiting both of these microenvironments. This effect is most pronounced in the communities of the Fe-rich nodule and related sediments (Fig. 5b).

The community richness (defined by the number of OTUs) and diversity (measured by the Shannon diversity index) are comparable for microbial populations of the Fe–Mn deposits and their underlying sediments at each of the three sites (Table S2).

The results of 16S rRNA gene sequencing have demonstrated that all of the analyzed microbial communities are predominated by *Pseudomonadota* of the *Kiloniellaceae* (1.62–8.21%), *Hyphomicrobiaceae* (1.30–6.73%), *Methyloligellaceae* (1.71–7.94%), and *Woeseiaceae* (0.57–8.26%) families, as well as planctomycetes of the *Pirellulaceae* family (Fig. 4). Additionally, proteobacteria of the *Rhodobacteraceae* (0.94–2.79%) and *Halieaceae* (0.10–4.32%) families, actinobacteria of the *Microtrichaceae* family (0.21–9.94%), crenarchaea of the *Nitrosopumilaceae* family (0.09–5.24%), as well as bacteria belonging to the *Flavobacteraceae* (0.38–3.17%, *Bacteroidota* phylum) and *Desulfosarcinaceae* (0.04–3.67%, *Thermodesulfobacteriota* phylum recently reclassified from *Deltaproteobacteria*) families, have rather high abundances in the majority of analyzed communities, but the representation of these groups varies between different samples (Fig. 4).

Different types of Fe–Mn deposits reveal clear differences in their community composition with 11 to 20% of taxa unique for each of the samples. The community of Fe-rich nodule is predominated by proteobacteria of the *Woeseiaceae* (3.39%) family, actinobacteria of the uncultured group 67–14 (2.32%), *Entotheonellaceae* (2.28%) and *Desulfuromonadaceae* (1.50%) representatives. Another Fe-rich microenvironment, the crust, harbors the microbial population specifically enriched with *Spongiibacteraceae* (3.71%), *Granulosicoccaceae* (2.72%) and *Ectothiorhodospiraceae* (1.63%) phylotypes, as well as the representatives of uncultured proteobacterial clade OM182 (1.21%). Some taxa have the highest abundance in the communities of both types of Fe-rich deposits. Those are the *Microtrichaceae* (2.17–9.94%), *Nitrospiraceae* (2.86–5.17%), *Cyclobacteriaceae* (0.88–4.13%), *Geopsychrobacteraceae* (1.43–3.88%) families. The community composition of the Mn-rich nodule is more dissimilar from that of Fe-rich deposits being specifically enriched with planctomycetes of the *Scalinduaceae* family (2.99%) and proteobacteria of the *Magnetospiraceae* (2.69%), *Nitrincolaceae* (1.01%), and *Thiohalorhabdaceae* (1.40%) families. Finally, two phylotypes, of bacterial *Kiloniellaceae* family and archaeal *Nitrosopumilaceae* family, are highly enriched in all the communities associated with the studied Fe–Mn deposits (Fig. 4).

Microbial communities of the underlying sediments are predominated by bacterial taxa abundant in all of the analyzed samples (*Pirellulaceae*, *Methyloligellaceae*, *Woeseiaceae* and *Hyphomicrobiaceae*) (Fig. 4). A large proportion of phylotypes have their highest abundances in the sediments. These are phylotypes belonging to *Thermodesulfobacteriota*, in particular, those of the *Desulfobulbaceae* (1.57–5.54%) and *Desulfosarcinaceae* (0.83–3.67%) families, and uncultured Sva1033 group (2.72–3.40%). In addition, all the sedimentary communities are enriched with the *Halieaceae* (2.22–4.32%), *Rhodobacteraceae* (1.31–2.79%), *Anaerolineaceae* (0.30–1.90%) and *Sandaracinaceae* (0.76–1.97%) phylotypes. *Sulfurovaceae* (10.21%), *Desulfocapsaceae* (5.21%)*,* and *Rubritaleaceae* (5.29%) are explicitly enriched in the underlying sediment from site AMK76-6236, while proteobacteria of the *Colwelliaceae* (1.71%) and *Sedimenticolaceae* (1.09%) families are enriched in the sedimentary community from site AMK76-6259 (Fig. 4).

**Figure 6:**
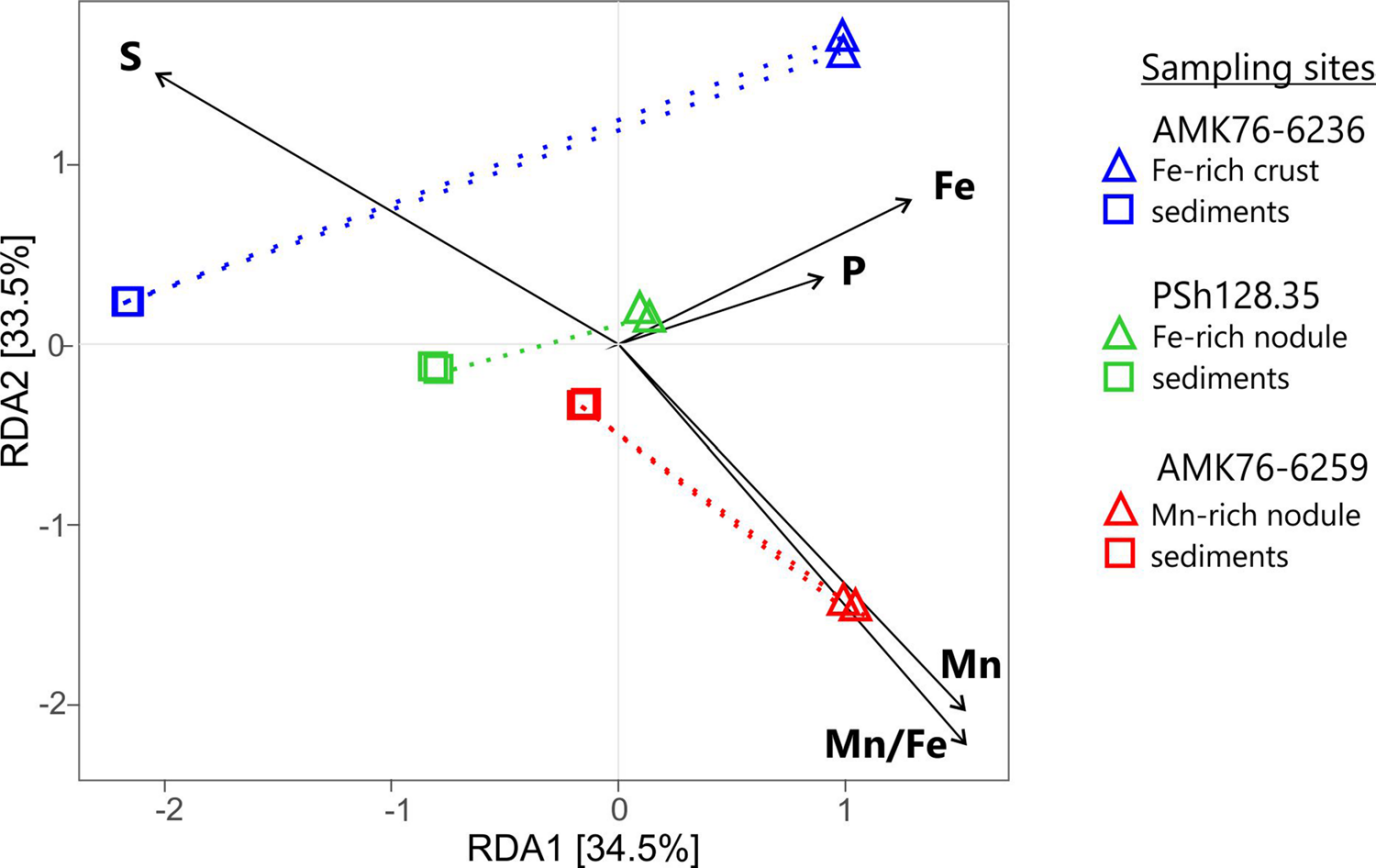
Redundancy analysis (RDA) showing the relationships between the compositions of major elements and microbial communities identified in the samples of Fe–Mn nodules, crust and underlying sediments. For visualization of RDA, the Rstudio software (R version 4.1.0) and microeco package were applied^77–79^.

The redundancy analysis (RDA) has revealed the response of the community composition on increased Fe or Mn content in the Fe–Mn deposits compared to the sediments (Fig. 6). The distribution of microbial communities of underlying sediments on the RDA plot do not correlate with the differences in the Fe or Mn content of these econiches, while the communities of nodules and crust, generally, line up on the plot in accordance with the elevated abundance of metals.

**Figure 7:**
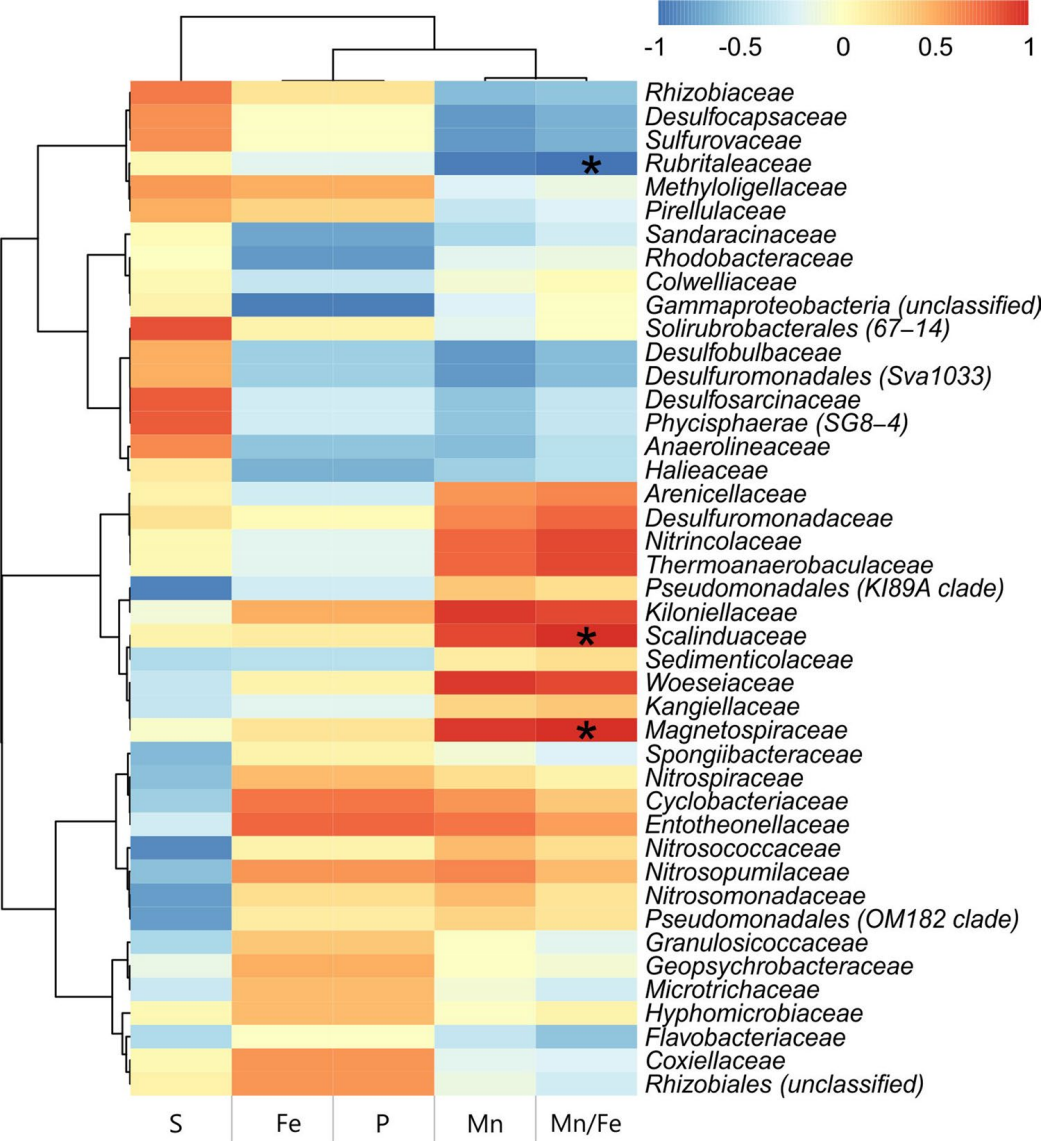
The correlation heatmap illustrates the relationships between relative abundances of dominating nodule- and sediment-associated families and geochemical parameters. The correlations were analyzed by Spearman’s rank correlation coefficient with Benjamini–Hochberg correction. For visualization of correlation analysis, the Rstudio software (R version 4.1.0) and microeco package were applied^77–79^.

The correlation analysis has demonstrated that the abundance of major elements (Fe, Mn, S, and P) noticeably affects the composition of microbial communities in all the sampled econiches (Fig. 7). The increase in *Magnetospiraceae*, *Scalinduaceae*, *Woeseiaceae* and *Kiloniellaceae* relative abundances correlates with the rise of Mn content, while high relative abundances of *Rhizobiales*, *Coxiellaceae*, and *Cyclobacteriaceae* positively correlates with the content of Fe and P. Sulfur content positively correlates with abundances of the taxa harboring sulfate reducers (*Desulfocapsaceae* and *Desulfosarcinaceae*) and sulfur oxidizers (*Sulfurovaceae*), and negatively correlates with abundances of widespread ammonia-oxidizing lithotrophs of the *Nitrosomonadaceae* and *Nitrosococcaceae* families.

## Discussion

### Kara Sea nodules and crust genesis

The main factors affecting the Fe–Mn deposits formation in the Kara Sea are relatively high sedimentation rate (2–45 cm/Kyr), enhanced amount of TOC in sediments (av. 1.18%), high input of terrestrial matter, and ice covering around nine months a year^29,30^. These environmental characteristics are reflected in the morphology and geochemistry of shallow-water nodules and crusts ^31^. Furthermore, the unstable organic matter (OM) supply, as well as bioturbation and physical mixing of the sediments by bottom current activity, contribute to the alternation of redox conditions in the sediments and porewater^32^. These variations can affect the fate of chemical elements involved in the formation of the Fe–Mn deposits. The sediments of the Kara Sea shelf are represented by reduced grayish muds covered by a thin (a few  cm thick) oxidized layer. Oxygenation of these sediments can lead to the precipitation of many metals including Fe and Mn, while O_2_ depletion in this sedimentary environment can mobilize metals^33,34^.

Our data revealed similar content of major elements in the nodules and crust collected at sites AMK76-6259, AMK76-6236, and PSh128.35 (Table 1). The elemental composition of the nodules collected at these sites appeared to be comparable with that previously described for the nodules from the central and eastern parts of the Kara Sea^35–37^ thus indicating the common chemical characteristics of Fe–Mn deposits from this shallow-water Arctic region. Manganese content of the studied samples is lower compared to that of deep-sea nodules (Clarion-Clipperton Zone, Peru Basin), while Fe content is generally higher than that reported for abyssal deposits^38^. All our samples of the deposits showed low Co + Ni + Cu content and highly variable Mn/Fe ratio. To classify Fe–Mn deposits, a triangle diagram by Bonatti ^39^ based on their chemical composition (Fe—Mn—(Cu + Ni + Co) × 10, Fe—Mn–Co × 100) is generally used. However, in case of the shallow-water fast-growing deposits this diagram does not work clearly. To date, the genesis of the Fe–Mn deposits has been characterized using various ratios of REY concentrations according to Bau^40^. Negative Ce anomaly, YSN/HoSN ratio close to 1, intermediate Nd concentrations of approximately 25 ppm suggest a strong impact of diagenetic processes on the formation of the studied Fe–Mn nodules and crust. The diagenetic origin of deposits in the other parts of the Kara Sea also was previously reported^35–37,41^. The observed depletion of major and trace elements (including REE) in the studied Kara Sea nodules and crust can be explained by their extremely high growth rate (0.4–8 mm/kyr) which results in dilution of authigenic mineral phases by detrital material due to short time of their connection with sediments and pore water^35,^. The intercalation observed between the Fe- and Mn-rich layers can occur due to differences in the normal redox potential of these elements and reflect the abrupt changes in their precipitation conditions. Redox oscillations in sediments of the Kara Sea occur periodically, or episodically, and vary between sampling sites^33,34^. Under temporal depletion of oxygen in the sediments, Mn and Fe can be remobilized and diffuse upwards. Mn-rich layers are usually formed in relatively steady bottom conditions (like those, prevailing at sampling site AMK76-6259 located on the inner shelf region with negligible freshwater inflow). The reduced sediments in this case are covered by a thin fluffy layer that acts as the upper oxidized one and prevents Mn outflow to seawater. Washing out of the fluffy layer due to dynamic bottom water circulation (that might take place at sampling site PSh128.35 on the outer shelf) could cause Mn transfer to the water (with its further carrying out by bottom currents) and initiate the formation of Fe-rich phases. This scenario of Fe-rich deposit formation is likely realized at site AMK76-6236 at the flank of the Saint Anna Trough, where the crusts lie on top of reduced sediments with low Mn content. The sea-floor in the trough is swept by intense bottom currents^42,43^.

The early diagenesis of nodules and crust can also be driven by the oxidation (remineralization) of OM. The input of OM to the Kara Sea bottom sediments, and as a consequence, to nodules and crust is strongly influenced by the river discharge, coastal erosion, sea-ice cover, and bottom circulation^44^. We observe the predominance of terrigenous OM in Mn-rich nodule, which presence is revealed by the unimodal distribution pattern of n-alkanes dominated by the C_27_, C_29_, and C_31_ odd homologues (Table S1). However, presence of bimodal n-alkane distribution in Fe-rich nodule indicates labile autochthonous OM (in moderate amount) along with terrigenous components. Accumulation of n-alkanes with different group composition of marine, bacterial-derived and terrigenous origin in the studied nodules and crust suggests diagenetic alteration of OM within the ore deposits. Odd–even ratio of n-alkanes (OEP_17-19_ < 1) suggests the OM undergoes an intense microbial degradation which could be coupled to biotic redox transformations of Fe and Mn minerals.

SEM images of the Kara Sea nodules showed a high abundance of different microfossils, similar to bacterial cocci and rods (Fig. 3) along with biofilm-like structures. These microfossils were identified in the interior of the Fe-rich and Mn-rich nodules. The same structures were previously observed in Fe–Mn nodules of the Pacific and Indian Ocean^11,20,45–47^. Well-crystallized Fe- and Mn-oxides around the microfossils indicate a post-accretional diagenetic process within the nodules. The same features were also observed in thin sections (Fig. 3i), where microbial cell-like structures are partially or completely filled with Fe- and Mn-containing minerals. Uniform thickness of ‘microbial cell coatings’ (0.2 µm) of the nodules indicates that fossilization of all the cells in the colony began simultaneously and was possibly caused by the exhaustion of organic carbon sources or hindered access to mineral electron acceptors (Fe and Mn-oxyhydroxides) due to the formation of surface thin films of their reduced forms upon microbial growth and dissimilatory metal reduction.

### Microbial communities of shallow-water Fe–Mn nodules and crust

An overview of relative abundance of prokaryotic taxa in microbial communities of the Kara Sea Fe–Mn deposits and their underlying sediments reveals a sufficient share of phylotypes common for all the analyzed econiches (Figs. 4, 5). Dominant taxa, in particular, *Pirellulaceae*, and alphaproteobacterial families *Kiloniellaceae*, *Hyphomicrobiaceae,* and *Methyloligellaceae*, have equally high representation in each of the analyzed samples (Fig. 4). This finding highlights general similarity of the microbial communities from the Kara Sea Fe–Mn deposits with previously reported communities associated with deep-sea or shallow-water Fe–Mn nodules. The majority of these diverse communities are dominated by *Alpha-* (e.g., *Rhizobiales*, *Kilonellales* and *Ricketsiales* orders) and *Gammaproteobacteria* (e.g., *Vibrionales* and *Xanthomonadales* orders), as well as by ammonia-oxidizing thaumarchaea of the *Nitrosopumilaceae* family and uncultured Marine Group I^11,13,14,16,24,25^. All these taxa include metabolically versatile organisms, which are capable of chemoorganotrophy or anoxygenic phototrophy^48^.

The distribution of unique OTUs between the communities of different Fe–Mn deposits from the Kara Sea indicates a divergence of these microbial populations from each other (Fig. 5). Each of the three microbial communities, associated with the Fe–Mn deposits, has its distinctive point on the RDA plot, according to the Fe or Mn content of its microenvironment (Fig. 6). The resulting picture indicates that the Fe and Mn abundance exerts selective pressure on the composition of the microbial populations inhabiting the nodules. Previously, a positive selection for *Gammaproteobacteria* of *Shewanella* and *Colwellia* genera was reported for deep-sea nodules from the Clarion-Clipperton Zone and South Pacific Gyre^13,14^. *Shewanella* is the model genus of metal-reducing bacteria^49^, and the *Colwellia* genus harbors Mn^4+^-reducers isolated from three different habitats^50^.

In the Kara Sea nodules, the microbial taxa, for which metal-reducing or -oxidizing activities were shown earlier, are represented by the *Geopsychrobacteraceae* family whose members are enriched in the nodules and crust from the sites AMK76-6236 and PSh128.35 (Fig. 4). These organisms belong to a recently proposed *Thermodesulfobacteriota* phylum^51^. Interestingly, a positive selection for another taxon of typical metal reducers (*Desulfuromonadaceae* family) was only observed in the Fe-rich nodule sample (Fig. 4). A more pronounced selection for Fe^3+^ reducers in the nodules might reflect a higher content and an amorphous state of Fe^3+^ minerals in this type deposits^35,37^, which makes them more readily accessible electron acceptors for iron reducing prokaryotes^52^. In addition to increased Fe availability, the Mn/Fe ratio of the Kara Sea nodules appeared to notably impact the abundance of metal-cycling organisms. The communities of the Mn-rich nodule sample (site AMK76-6259) have increased shares of *Magnetospiraceae* and planctomycetes of ‘*Scalinduaceae*’ family (Fig. 4) which were poorly represented in the rest of the analyzed samples (Fig. 7). None of these taxa comprise organisms utilizing Mn compounds as electron acceptors or donors for growth. Instead, magnetospirilla are known for their unique capability to produce fine-grained intracellular magnetite crystals, while ‘*Scalinduaceae*’ are capable of Fe^2+^ oxidation^53^.

Visualization of microcolonies-like structures inside the studied nodules (Fig. 3i) indicates that these deposits were formed under the influence of microbial biofilm growth and concomitant processes of exopolysaccharides production and acidification. Indeed, sponge symbionts of the *Entotheonellaceae* family, well-adapted to colonize the extended surfaces, were significantly selected for in Fe-rich nodules (Figs. 4, 7). Besides, the microbial communities of Fe-rich nodules and crust are enriched with actinobacteria of the *Microtrichaceae* family (Fig. 4) harboring filamentous heterotrophic species^54^. Within complex biofilms, fermentative bacteria of the *Hyphomicrobiaceae* family, abundant in the Kara Sea nodule communities (Fig. 4), could induce acidification by the production of fatty acids and thus, influence the mobility of Fe and Mn species and their susceptibility to further microbial redox transformations. Ratio Pr/Ph < 1 suggests reduced, low oxygen conditions in the samples from sites AMK76-6236 and AMK76-6259. Such microenvironments favor the microbial reduction of Fe^3+^ and Mn^4+^ minerals followed by their mobilization from the nodule matrix (Fig. 2). Nodules' organic matter can be degraded within the nodule microbial communities by bacteroidetes of the *Flavobacteriaceae* and *Cyclobacteriaceae* families, providing the substrates for predominating fermentative bacteria of the *Woeseiaceae* and *Hyphomicrobiaceae* families (Fig. 4), and for anaerobic organotrophs of the *Geopsychrobacteraceae* and *Desulfuromonadaceae* families capable of dissimilatory metal reduction.

In addition to direct involvement in redox cycling of Fe and Mn compounds, microbial populations of ferromanganese deposits could drive the biogeochemical cycle of nitrogen including the processes coupling the transformation of nitrogen and iron compounds. Indeed, the representatives of the *Kiloniellaceae* family prevailing in all the Kara Sea deposits (Fig. 4) were reported to couple nitrate reduction to organic matter oxidation. Representatives of archaeal *Nitrosopumilaceae* and bacterial *Nitrospiraceae* families, ubiquitous in almost all the sampled deposits (Fig. 4), can be involved in nitrification^17,55^. In addition, *Nitrospiraceae* representatives, which are mostly abundant in Fe-rich deposits (from sites PSh128.35 and AMK76-6236; Figs. 4, 7) can oxidize ammonia via comammox. In the Mn-rich nodule from site AMK76-6259, ammonium can be oxidized by planctomycetes of the *‘Scalinduaceae’* family^56,57^, which are enriched in this microenvironment (Fig. 4).

Ammonium concentration in the Kara Sea is generally low (micromolar range)^58,59^, and the selective pressure for ammonium oxidizers in this environment needs further evaluation. Previously, the enrichment of ammonia-oxidizing archaea of the *Nitrosopumilaceae* family was reported for several deep-sea nodules^11,13,14,16,27^. This taxon is globally abundant in marine environments and comprises autotrophs^60^, many of which depend on Fe availability for their growth^61,62^. Such physiological features could enhance the preferred colonization of growing ferromanganese nodules with these Fe-scavenging prokaryotes. Thus, the growth and metabolic activity of ammonia oxidizers could impact the Mn/Fe ratio during diagenetic processes occurring within the nodules. In its turn, ammonium oxidation in the oxygenated surface layer of nodules could be fueled by ammonium release from organic matter degradation in the water column or underlying sediments^63,64^ or within the biofilms of fermentative bacteria inside the Fe–Mn deposits. In addition, ammonium can be formed via dissimilatory nitrate reduction to ammonium (DNRA). This process was shown for *Nitrincolaceae*, enriched in the Mn-rich nodule of site AMK76-6259 (Figs. 4, 7). In the Fe-rich nodule, nitrate reduction can be also coupled to the oxidation of Fe^2+^ (NRFeOx). This process is proposed to be common for mixotrophic denitrifiers^65,66^, including those of the *Hyphomicrobiaceae* family with rather high abundance in Fe-rich sites PSh128.35 and AMK76-6236 (Fig. 4).

### Microbial communities of sediments underlying ferromanganese deposits

The microbial communities identified in shallow-water Kara Sea sediments appeared to be depleted with the taxa potentially involved in nitrogen cycling, when compared to the communities of Fe–Mn deposits from the same sites. This effect is clearly evidenced by decreased abundance of potential ammonia-oxidizers (harbored by the *Nitrosomonadaceae* and *Nitrosococcaceae* families) in the sampled sediments (Fig. 4). The decreased representation of ammonia oxidizers could result from the peculiarities of OM inflow regime, observed at the sampling sites. Unstable inflow of OM, temporal variation of redox gradient and local formation of anoxic niches in the sediments exert selective pressure on sedimentary microbial communities towards the prevalence of anaerobically respiring organisms. Comparatively low abundance of nitrogen cycling microorganisms was previously reported for Baltic Sea sediments, although the diversity of these organisms appeared to be rather high and included the nitrifying archaea of *Nitrosopumilales* order, various nitrifying, denitrifying and DNRA performing bacteria^67^. Obviously, high anthropogenic activity and local hydrodynamics significantly contribute to nitrogen input and the intensity of its biological transformation in the Baltic Sea region^67^, that is not the case for the Kara Sea, subjected to incomparably lower input of nitrogen compounds. All the benthic microbial communities in our study are dominated by the taxa capable of sulfate respiration (*Desulfosarcinaceae* or *Desulfocapsaceae* families) (Fig. 4). The abundance of these taxa positively correlates with the abundance of sulfur in the environment (Fig. 7). RDA analysis clearly separated the community of sulfur-enriched sediments from the others (Fig. 6), and the 16S rRNA gene sequence abundance supported positive selection for sulfate reducers in this microenvironment (Fig. 4). High relative abundance of the orders *Desulfobacterales, Desulfovibrionales, Desulfuromonadales*, which include a lot of sulfate reducing bacteria, was reported for the sediments of the Yamal Sector of the Kara Sea^68^. Also, *Desulfobulbaceae, Desulfuromonadaceae*, and *Pelobacteraceae* were found to be enriched in the sediments of the North Sea and Baltic Sea near the Fe(III)/Fe(II) redox boundary, while higher abundance of *Desulfococcus* and *Desulfobacterium* was detected at greater depths of these sediments with decreased Fe-reducing activity^69^. All these taxa harbor a wide variety of both Fe reducing, and sulfate reducing bacteria. In our study, we did not detect the enrichment of any typical metal reducing organisms in benthic microbial communities, except for the sediments underlying Mn-rich nodules, where phylotypes belonging to organotrophic Mn^4+^-reducing *Colwelliaceae* were enriched. In addition, anaerobically respiring organotrophs of the *Halieaceae* family and fermentative organotrophs belonging to *Anaerolineaceae* are enriched in almost all the benthic communities (Fig. 4). The increased abundance of *Rhodobacteraceae*-related phylotypes in sedimentary microbial communities could play a crucial role in sulfur and carbon biogeochemical cycling under fluctuating redox conditions of the Kara Sea sediments, as this family comprises aerobic phototrophs, chemoheterotrophs, and anoxygenic phototrophs which utilize hydrogen sulfide as the electron donor^48^. The benthic microbial community at site AMK76-6236 is highly enriched with *Sulfurovaceae*-related phylotypes (Fig. 4). Relatively low but considerable (ca. 1%) abundance of *Sulfurovaceae* was previously reported for the sediments of the Yamal Sector of the Kara Sea^68^. Members of this family are chemolithoautotrophs coupling sulfur oxidation with nitrate reduction or oxygen respiration, which makes them crucial primary biomass producers in the community at both oxic and anoxic conditions. The increased representation of aerobic marine oligotrophs of the *Rubritaleaceae* family in the same benthic community of site AMK76-6236 correlates with its higher oxygenation compared to the other two sampling sites. The oxygenation of the bottom water in this deep part of the St. Anna Trough is driven by the cooling of oxygen-rich surface waters near the Novaya Zemlya archipelago and their further downwelling across the slope^58^. During the temporal decrease of oxygenation at this site, reduced sulfur species can be produced by sulfate-reducing bacteria of the *Desulfobulbaceae*, *Desulfosarcinaceae* and *Desulfocapsaceae* families, some of which were reported to establish single-species filamentous conductive structures (‘cables’), which couple the oxidation of sulfide in deeper sediment layers to the reduction of oxygen or nitrate near the sediment–water interface^70^.

The enhanced abundance of microorganisms capable of respiring a variety of electron acceptors in the Kara Sea sediments correlates with unstable redox conditions of this environment. The sharp changes of the redox state due to fluctuating input of organic matter and oxygenated waters seem to be the main selective force making the Kara Sea sediments the reservoir of metabolically versatile cosmopolitan marine prokaryotes involved in the biogeochemical cycles of carbon and sulfur, as well as in redox cycling of metals. The cumulative geochemical activity of such a diverse microbial community is expected to follow the changes in environmental conditions, which periodically favor the predominance of different metabolic groups of microorganisms. We have shown that comparatively stable redox and geochemical settings within the semi-closed ferromanganese deposits directly select for the organisms which reduce, oxidize or intracellularly accumulate metals. The overall metabolic activity of these microorganisms, sheltered in nodules and crust from unfavorable fluctuations of environmental factors, is expected to dramatically accelerate the formation of the Fe–Mn deposits different in their morphology, internal structure and elemental composition.

## Methods

### Field sampling

Fe–Mn nodules, crust and underlying sediments were collected during the research cruises AMK-76 and PSh128 in 2015 and 2019. Samples were collected from three different regions of the Kara Sea with different environment conditions: open sea (site PSh128.35, depth 140 m), inner shelf region with negligible freshwater inflow, west of the Yamal Peninsula (site AMK76-6259, depth 91 m) and a trough with terrigenous matter input from Franz Josef Land and the Barents Sea via Atlantic water inflow (site AMK76-6236, depth 237 m)^34,44^. Nodules and crust samples were collected using a trawl. Coordinates and depths of the sampling sites are given in Table S4. Based on the size and morphology of the nodules, the dominant nodule type was determined at each site. Nodules corresponding to the dominant type, as well as the crust were taken out of trawls with sterile tweezers, gently rinsed with seawater, and put into sterile plastic bags, which were than sealed, frozen at − 21 °C, and transported to the laboratory at this same temperature. These samples were used for geochemical and organic matter investigations. Samples for DNA extraction were stored at the above-mentioned conditions for 1 month prior to analysis.

Samples of underlying sediments (0–2 cm) were obtained at each of the trawling sites using the boxcorer. All the sediment samples were taken out with pre-sterilized stainless steel spatula into sterile plastic bags and further stored similar to the nodules and crust before analysis.

DNA for sequencing was extracted from six samples, two from each of the three sampling sites (2 nodules, 1 crust, and 3 sediment samples were totally subjected to DNA extraction). Geochemical and organic matter studies were carried out on another six samples (2 nodules, 1 crust, 3 sediment samples) from all the three sampling sites.

### Samples description

Fe–Mn nodules from sites AMK76-6259 and PSh128.35 are discoidal in shape, have diameter up to 10 cm with thickness of up to 1.5 cm and display morphological features of growth in a horizontal direction from the center to the rim (Fig. 1). Fe–Mn crusts from site AMK76-6236 are irregular in shape, 4–12 mm-thick, their surface is concave with holes filled with sediments. Underlying sediments are mainly presented by terrigenous silty sand (AMK76-6258, PSh128.35) and sandy silt (AMK76-6236).

### Geochemistry

The bulk nodules (2 samples), crust (1 sample) and underlying sediments (3 samples) were freeze-dried, ground, homogenized, and analyzed for the content of major and some trace elements (Na, Mg, P, S, K, Ca, Al, Ti, V, Cr, Mn, Fe, Co, Ni, Cu, Zn, and Sr) by ICP atomic emission spectrometry (ICP-AES) (ICAP-61, Thermo Jarrell Ash, USA). The trace-element (Li, Sc, Cr, Ni, Cu, Zn, Sr, Mo, Cd, Ba, W, and Pb) concentrations of the samples listed above were determined by ICP-MS (X-7, Thermo Elemental, USA). Samples were dried, crushed to powder size and dissolved with HNO_3_ + H_2_O_2_ (4:1 by volume, Merck) in an autoclave system. The accuracy of the measurements was 3–5%. Analytical precision and accuracy were checked by analyses of standards OOPE201 (SDO-2) and OOPE601 (SDO-4). A detailed description of the methodology and accuracy is given in Karandashev et al.^71^.

### Scanning Electron Microscopy (SEM–EDS)

Nodules were soaked with low viscosity epoxy in a vacuum before and between cutting. The most representative areas, up to 25 × 45 mm, were selected and then thin sections (about 200 µm thick) were made for SEM investigation. Backscattered electron (BSE) imaging, secondary electron (SE) imaging and energy dispersive X-ray spectroscopy (EDS) analyses were performed using Tescan scanning electron microscope Mira 3 with analytical equipment of the Oxford Instruments AztecLive Automate with detector Max 80. The SEM was operated at 10 kV for BSE images, 4 kV for SE images and 20 kV for EDS analyses. Sections were subsequently mapped in Si, Mn, Fe, Ca and P X-rays using the Aztec program. Si, Mn, Fe, Ca and P were measured using Kα lines. Investigations of the nodule microstructures were made by SEM analyses with a Mira 3 TESCAN. Polished thin, 150 μm-thick sections were prepared. Fragments of the outer parts and central zone of nodules were selected for the identification of microbial associated structures. The surface of the samples was covered with 15 nm-thick gold with Balzers SCD 030 equipment. Major element concentrations were determined using energy-dispersive X-ray spectroscopy on an X-MAX 80 (EDS, Oxford Instruments, UK). Analyses were conducted at 20 kV using a diaphragm of 60 μm. Data analysis was carried out using the INCA Oxford software package.

### Bulk analyses

Nodules, crust, and sediment samples were frozen onboard at − 21 °C prior to freeze-drying on land. Dried samples were ground in a mortar. Total organic carbon (TOC) was measured on a Shimadzu L-VPH. TOC was determined on decalcified (acidification with 10% HCl) samples. The instrument was calibrated using standards SDO-2 for sediments and SDO-4 for nodules^72^.

### GC–MS

For the n-alkane analyses, the nodule, core and sediment samples were stored at -21 °C directly after collecting. The freeze-dried samples (10–20 g) were ground, homogenized, and subjected to extraction under ultrasonication with dichloromethane:methanol mixture (9:1). The total extract was purified from sulfur using activated copper. The n-alkanes were separated from the other fractions by liquid chromatography on silica gel with hexane as the eluent. GC–MS analyses were performed using a Shimadzu QP5050 using a Rxi-5Sil MS 30 m × 0.25 mm × 0.25 µm capillary column. The temperature program was as follows: starting with 3 min at 60 °C, then heating to 300 °C at 4 °C/min, and then holding 30 min at 300 °C. The injection volume was 2 µl, splitless. Carrier gas was helium with a flow rate of 1.5 ml/min. The analysis was made as a total scan from m/z 50 to 650 (70 eV). Identification and quantification of n-alkanes were made on the basis of the retention times of the calibration mixture (n-C_8_-C_20_, n-C_21_-C_40_ mixtures, Fluka). Response factors were determined relative to squalane (2, 6, 10, 15, 19, 23-hexamethyltetracosane) as the internal standard. Concentrations of individual hydrocarbons were reported in μg/g of the dry sample.

### DNA extraction and 16S rRNA gene sequencing

DNA was isolated from the frozen samples of nodules, crust and sediment using the commercial FastDNA™ Spin Kit for Soil (MP Bio, Salt Lake City, UT, USA) according to the manufacturer’s instructions. Subsamples of ca. 0.5 mL bulk volume were taken for DNA extraction. Ferromanganese deposits were preliminary ground into a paste with sterile tweezers and spatula. Preparation of amplicon libraries of the V4 region of the 16S rRNA gene for Illumina MiSeq high-throughput sequencing and the primer system including Illumina Linker Sequences, Indices, Heterogeneity Spacers and 515/806 primer sequences, were performed as previously described^73^. The primary processing of the raw reads was performed as described earlier ^74^. All the reads of the V4 region of 16S rRNA gene were analyzed using the SILVAngs service with default parameters (https://ngs.arb-silva.de/silvangs/) and SILVA138.1 SSU database.

### Statistical analysis

The diversity of microbial communities (measured by the Shannon diversity index) was evaluated by the Kruskal–Wallis test^75^ with Benjamini–Hochberg correction^76^. Statistical analyses were performed in R 4.1.0 using the p.adjust function^77,78^. The RDA was used to assess the response of the community composition to the variations of geochemical parameters within Fe–Mn deposits and underlying sediments. The correlation between relative abundances of identified families and geochemical parameters was analyzed by Spearman's rank correlation coefficient with Benjamini–Hochberg correction^76^. The geochemical data are available in Table 1. For visualization of RDA and correlation analysis, the Rstudio software (R version 4.1.0) and microeco package were applied^77–79^.

## Supplementary Information

Supplementary Information.

## Data Availability

The datasets of chemical parameters (element and hydrocarbon compositions) and gene sequences related to the studied nodules, crust and underlying sediments are attached as supporting information (Tables S1–S4). All the obtained sequences were deposited into NCBI under the BioProject accession number PRJNA758033.
